# Supplementary material for: Mosaic pattern formation in exfoliated graphene by mechanical deformation
Source: Nat Commun. 2019 Apr 5;10:1572. doi: 10.1038/s41467-019-09489-z (PMC6450902; doi:10.1038/s41467-019-09489-z)
Supplement: Supplementary file 1 — Supplementary Information [file 41467_2019_9489_MOESM1_ESM.pdf]

Supplementary Information

Mosaic pattern formation in exfoliated graphene by mechanical deformation

Maria Giovanna Pastore Carbone *et al.*

Supplementary Discussion

The origin of mosaic formation emanates from the combination of two phenomena i.e. the wrinkling of graphene under tensile (lateral direction) and compressive (axial direction) mechanical deformation. However, the latter appears due to the gradual relaxation of tensile strain resulting from interface slippage. The exact strain for which this is triggered depends on the interface shear strength between graphene and polymer and its threshold value depends on a number of parameters as will be explained below. These phenomena acting independently (axial or lateral wrinkling or buckling) on graphene simply supported on PMMA substrates have already been examined in recent works of the group and the corresponding critical strains for buckling were found experimentally and predicted theoretically. In general, several theoretical models have been adopted to predict the critical strain for buckling for thin sheets on substrates[1,2,3]; in two recent works of our group[4,5,6], wrinkling of graphene supported on polymeric substrate under compression and lateral wrinkling were predicted by combining the Euler mechanics with a Winkler approach and the interaction with the substrate simulated with linear elastic springs, thus yielding:

$$\varepsilon_{cr}^{comp} = \pi^2 \frac{D}{C}\frac{k}{w^2} + \frac{l^2}{\pi^2 C}\left(\frac{K_w}{m^2}\right) \qquad (1)$$

$$\varepsilon_{cr}^{tens} = \frac{\pi^2 D(\frac{m^2}{l^2}+\frac{n^2}{w^2})^2}{C(\nu\frac{n^2}{w^2}-\frac{m^2}{l^2})} + \frac{K_w}{\pi^2 C(\nu\frac{n^2}{w^2}-\frac{m^2}{l^2})} \qquad (2)$$

where w and l are flake dimensions, $k = \left(\frac{mw}{l} + \frac{lm}{w}\right)^2$, m and n are the half waves in x and y direction, D is the bending rigidity, K_w is the Winkler's modulus and ν is the Poisson's ratio of the substrate. By solving eq. 1 and 2 for 1LG flake of dimensions larger than critical lengths on PMMA, ε_{cr}^{comp} and ε_{cr}^{lat} have been found to be, respectively, ~0.3% and 1.2%; For 2LG on PMMA, ε_{cr}^{comp} and ε_{cr}^{lat} have been found to be, respectively, ~0.2% and 0.6%[4,5,6]. It is

also interesting noting that – according to these models – the density of wrinkles for 1LG is higher than 2LG.

Regarding the interface strength, it is well known that graphene/polymer interface is dominated by van der Waals forces and values of interfacial shear strength ranging from 0.3 to 0.8 MPa[7,8] have been measured by the combination of mechanical tests and Raman spectroscopy. Sliding in graphene/polymer systems has in general been found to occur at a critical level of applied deformation that, for polymeric matrices such as PET and PMMA, initiates at around 1.2-1.5%[9].

In order to analyse the combined mechanism of wrinkling and interface slippage, let's consider a typical plot (Figure 4e) of strain in graphene versus strain applied to the substrate during a loading-unloading cycle. During loading, when the maximum interfacial shear stress reaches its maximum attainable value, interfacial sliding occurs starting from the edges of the flakes[9] and, at a certain level of applied deformation (ε_p), the strain in graphene eventually saturates at a constant level. Briefly, ε_p is the maximum strain that can be transferred from polymer substrate to graphene and this value has been theoretically estimated[9] as

$$\varepsilon_p = \frac{\tau_c l}{2C} \qquad (3)$$

where τ_c is the interfacial shear strength, l is the length of the flake and C is the 2D Young's modulus (tension rigidity) of monolayer graphene.

If the maximum deformation applied to the substrate (indicated in the plot as ε_m) is higher than ε_p, then, no additional increment of strain can be transmitted to the graphene flake due to interfacial slippage. Upon unloading at a certain critical point, ε_0 ($\varepsilon_0 = \varepsilon_m - \varepsilon_p$) the graphene flake will be subjected to compressive strain (see Figure 4 in the main text). When the induced compressive strain exceeds the critical strain for wrinkling ε_{cr}^{comp}, then compression-induced (transverse) wrinkles will appear.

Hence, it can be postulated that the applied strain level at which mosaic originates $\varepsilon_{onset}^{mosaic}$ is

$$\varepsilon_{onset}^{mosaic} = \varepsilon_m - (\varepsilon_p + |\varepsilon_{cr}^{comp}|) \qquad (4)$$

By substituting equations (1) and (3), equation (4) yields

$$\varepsilon_{onset}^{mosaic} = \varepsilon_m - \frac{\tau_c l}{2C} - \pi^2 \frac{D}{C}\frac{k}{w^2} + \frac{l^2}{\pi^2 C}\left(\frac{K_w}{m^2}\right) \qquad (5)$$

Since, for monolayer graphene supported on PMMA the ε_{cr}^{comp} has been experimentally found equal to 0.3%[6] and ε_p around 1.5%, it is now possible to predict the minimum applied strain required for the formation of mosaic morphology. This minimum applied strain is thus $\varepsilon_p + |\varepsilon_{cr}^{comp}|$~1.8 % (see Supplementary Figure 2).

Further evidence of the gradual release of compressive strain after buckling has been finally verified by independent mechanical experiments combined with Raman spectroscopy, as shown in Figure 4e,f. We have to underline at this point that the critical tensile strain for out-of-plane lateral buckling is very sensitive to the level of adhesion and therefore its value will depend on (a) the strength of the graphene/ polymer bond (b) possible surface modification of graphene (c) chemical (bulk) modification of the substrate and (d) strain rate effects.

Supplementary Methods

Tensile test of neat PMMA

Uniaxial tensile test of PMMA adopted in this work has been performed according to ASTM D638 – 14.

Multiple loading/unloading

Cyclic loading/unloading experiments were performed on bilayer graphene: the specimen was subjected to tensile tests of 20 loading-unloading cycles at three different deformation levels (0.1%, 0.2% and 0.3%). AFM images have been acquired before and after each multiple loading/unloading cycle.

Control test on PMMA substrate

We have performed the control test by acquiring AFM images from the substrate upon loading and unloading. As shown in the Supplementary Figure1, no change of the roughness can be observed (R_a is approximately 0.5 nm) hence we are confident that the formation of wrinkles in graphene does not come from any undulations of the surface of the substrate.

Supplementary Figures

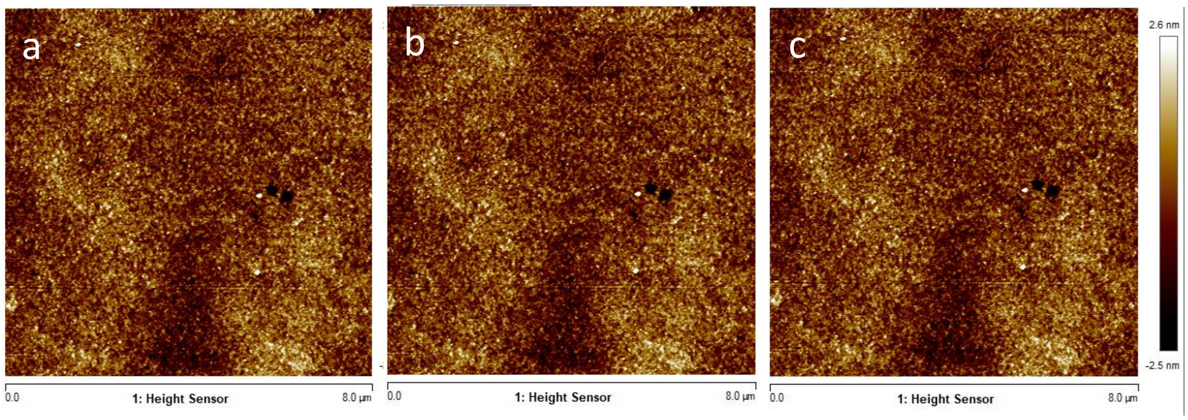

Supplementary Figure 1. Control test on PMMA substrate: AFM images of PMMA substrate at rest (a), upon application of tensile deformation 1% (b) and after unloading (c).

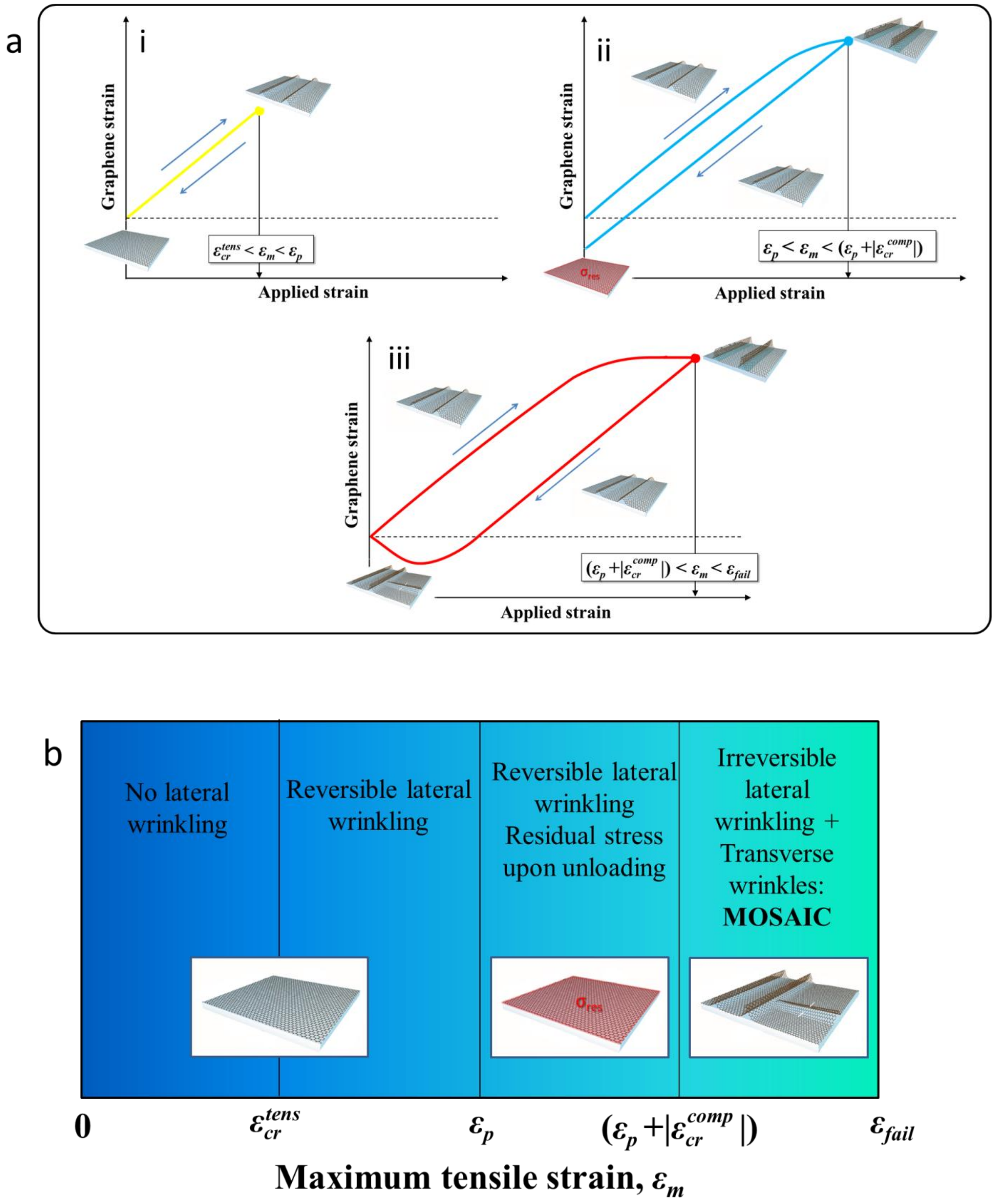

Supplementary Figure 2 Supported graphene upon loading-unloading: typical cycles with increasing maximum applied strain (a) and synoptic diagram of final morphologies (b).

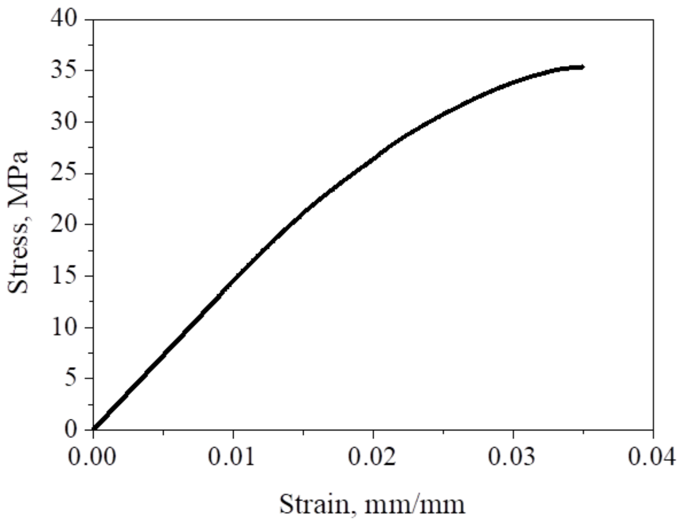

Supplementary Figure 3. Stress/strain curve of the PMMA adopted in this study

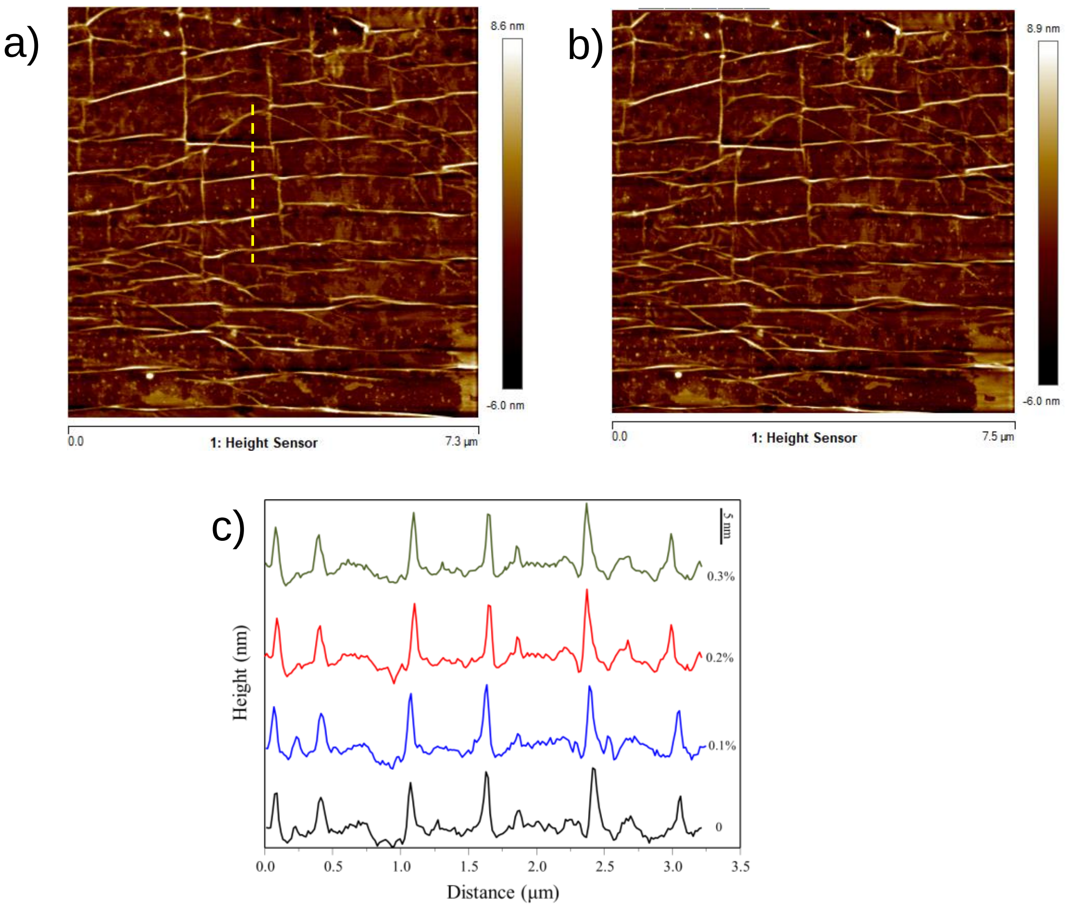

Supplementary Figure 4. Multiple loading/unloading cycles on mosaic: AFM image of the mosaic morphology in the starting configuration (a) and after 20 loading/unloading cycles at 0.3% (b). Height profiles for the starting morphology and after 20 cycles at 0.1%, 0.2% and 0.3% (the cross-section line is indicated in the AFM image). It is evident that the mosaic morphology remains unaltered after multiple cycles within the investigated deformation range.

Supplementary References

1. Huang, Z. Y., Hong, W. & Suo, Z. Ã. Nonlinear analyses of wrinkles in a film bonded to a compliant substrate. *J. Mech. Phys. Solids* **53,** 2101–2118 (2005).

2. Vella, D., Bico, J., Boudaoud, A., Roman, B. & Reis, P. M. The macroscopic delamination of thin films from elastic substrates. *Proc. Natl. Acad. Sci.* **106,** 10901–10906 (2009).

3. Pan, K., Ni, Y., He, L. & Huang, R. Nonlinear analysis of compressed elastic thin films on elastic substrates: From wrinkling to buckle-delamination. *Int. J. Solids Struct.* **51,** 3715–3726 (2014).

4. Androulidakis, C. *et al.* Failure processes in embedded monolayer graphene under axial compression. *Sci. Rep.* **4,** 1–8 (2014).

5. Androulidakis, C., Koukaras, E. N., Pastore Carbone, M. G., Hadjinicolaou, M. & Galiotis, C. Wrinkling formation in simply-supported graphenes under tension and compression loadings. *Nanoscale* **9,** 18180–18188 (2017).

6. Koukaras, E. N., Androulidakis, C., Anagnostopoulos, G., Papagelis, K. & Galiotis, C. *Compression behavior of simply-supported and fully embedded monolayer graphene: Theory and experiment*. *Extreme Mechanics Letters* **8,** (2016).

7. Anagnostopoulos, G. *et al.* Stress Transfer Mechanisms at the Submicron Level for Graphene/Polymer Systems. *ACS Appl. Mater. Interfaces* **7,** 4216–4223 (2015).

8. Gong, L. *et al.* Interfacial stress transfer in a graphene monolayer nanocomposite. *Adv. Mater.* **22,** 2694–2697 (2010).

9. Jiang, T., Huang, R. & Zhu, Y. Interfacial sliding and buckling of monolayer graphene on a stretchable substrate. *Adv. Funct. Mater.* **24,** 396–402 (2014).
